# Nasal Microbiome Change During and After Exacerbation in Asthmatic Children

**DOI:** 10.3389/fmicb.2021.833726

**Published:** 2022-03-04

**Authors:** Tsunglin Liu, Cheng-Han Lin, Yi-Lin Chen, Shuen-Lin Jeng, Hui-Ju Tsai, Chung-Liang Ho, Wen-Shuo Kuo, Miao-Hsi Hsieh, Pei-Chi Chen, Lawrence Shih-Hsin Wu, Jiu-Yao Wang

**Affiliations:** ^1^Department of Biotechnology and Bioindustry Sciences, National Cheng Kung University, Tainan, Taiwan; ^2^Molecular Diagnostic Laboratory, Department of Pathology, National Cheng Kung University Hospital, Tainan, Taiwan; ^3^Department of Statistics, Center for Innovative Fin Tech Business Models, Institute of Data Science, National Cheng Kung University, Tainan, Taiwan; ^4^Institute of Population Health Sciences, National Health Research Institutes, Zhunan, Taiwan; ^5^Center of Allergy, Immunology, and Microbiome (AIM), China Medical University Children’s Hospital, Taichung, Taiwan; ^6^Graduate Institute of Biomedical Sciences, China Medical University, Taichung, Taiwan; ^7^Allergy and Clinical Immunology Research (ACIR) Center, National Cheng Kung University, Tainan, Taiwan; ^8^Department of Allergy and Immunology, China Medical University Children’s Hospital, Taichung, Taiwan

**Keywords:** childhood asthma, acute exacerbation, mite allergy, nasal microbiota, recovery phase

## Abstract

Airway and gut microbiota are important in asthma pathogenesis. Although several studies have revealed distinct microbiota in asthmatic airways at baseline compared to healthy controls, limited studies compared microbiota during acute exacerbation (AE) and in the recovery phase (RP) in the same asthmatic children. We aim to investigate association between microbiota and asthma status in children and explore their relationship with clinical features of asthma. We recruited 56 asthmatic children and investigated their nasal, throat, and stool microbiota during AE and in the RP. Totally, 320 samples were subjected to 16S rRNA sequencing. Although the microbial communities were clearly separated by body site, within each site the overall communities during AE and in the RP could not be distinguished. Most nasal microbiota were dominated by only one or two of six bacterial genera. The domination was associated with mite allergy and patient age only during AE but not in the RP. When moving into RP, the relative abundance of *Staphylococcus* increased while that of *Moraxella* decreased. Throat and stool microbiota were not associated with most of the clinical features. Interestingly, stool microbiota during AE was associated with ABO blood type and stool microbiota in the RP was associated with frequency of the subsequent exacerbations. In summary, the association between nasal microbiota and mite allergy only during AE suggests an altered local immunity and its interplay with nasal microbes. Our work provides a basis for studying microbes, and prevention or therapeutic strategy in childhood asthma, especially during AE.

## Introduction

Pediatric asthma is the most common chronic disease in wealthy countries, and its prevalence is increasing in developing countries (Martinez and Vercelli, 2013; Pawankar, 2014). The etiology of asthma is complex, and many studies have indicated genetic and environmental factors (Friedman and Zeiger, 2005; Renz-Polster et al., 2005; Kozyrskyj et al., 2007; Wang et al., 2013; Thomsen, 2015), such as exposure to allergens and microbes, especially in early childhood. For example, childhood asthma has been associated with the mode of delivery (Renz-Polster et al., 2005), breast-feeding (Friedman and Zeiger, 2005), and antibiotic use (Kozyrskyj et al., 2007; Wang et al., 2013). These studies lead to a concept that early life alteration of gut microbiota shapes the immune system, thereby affecting asthma development (Budden et al., 2017). Other environmental factors, such as growing up in farm (Ege et al., 2011; Heederik and von Mutius, 2012), pet ownership (Ownby et al., 2002), living with older siblings, or early daycare attendance (Ball et al., 2000), have also been reported. These indicate that airway exposure to allergens or microbes can modulate immune system and increase risk of developing asthma. Studying airway microbes, Bisgaard et al. (2007) showed that the colonization of *Streptococcus pneumoniae*, *Moraxella catarrhalis*, and/or *Haemophilus influenzae* in the throats of infants at the first month of age was associated with persistent wheeze in the first 5 years of life and diagnosis of asthma by age 5. Pathogenic association between the early life airway bacteria and childhood asthma was also reported in later studies using high-throughput sequencing (Teo et al., 2015; McCauley et al., 2019; Zhou et al., 2019).

Although several studies have investigated airway or gut microbiota in childhood asthma (Bisgaard et al., 2007; Arrieta et al., 2015; Cui et al., 2015; Teo et al., 2015, 2018; Bomar et al., 2018), limited studies examined nasal microbiota in school children and adolescents (Kloepfer et al., 2014; Perez-Losada et al., 2017; McCauley et al., 2019; Zhou et al., 2019), and only two focused on acute exacerbation (AE) (McCauley et al., 2019; Zhou et al., 2019). AE represents a specific window of time of an agitated immune system. Simultaneous investigation of airway and gut microbiota in asthmatic children is also scarce. Moreover, following microbiota of the same patients alleviates the concern of individual differences, but is not commonly implemented. Here, we collected airway and stool samples from 56 asthmatic children and adolescents during AE and in the recovery phase (RP). The microbiota and changes were examined *via* 16S rRNA sequencing, and their associations with clinical features were studied.

## Materials and Methods

### Patient Recruitment

Fifty-six asthmatic patients at age 3–17 with recurrent wheeze were recruited prospectively between January and May 2018. A patient without fever and visiting outpatient clinics or emergency department was diagnosed as AE of asthma if he/she had an acute or sub-acute episode of progressive worsening of symptoms, such as shortness of breath, wheezing, cough, chest tightness, and needing acute reliever treatments. Patients in RP (approximately 2 weeks after AE) was defined as physician-diagnosed asthma, presenting for routine, non-urgent, and asthma follow-up care. We excluded patients having an AE with fever and/or been treated with antibiotics, immunotherapy, oral or parenteral corticosteroids administered for more than 15 consecutive days, depot steroids, inhaled corticosteroids (beclomethasone dipropionate) in doses greater than 1,000 μg/day, or inhaled β2-agonists more than four times a day (severe asthma).

### Ethical Approval and Consent to Participate

This study protocol was approved by the Ethical and Clinical Trial Committee and the investigation review board of National Cheng Kung University Hospital (NCKUH) (A-BR106-069). All patients’ caregivers provided written informed consent.

### Clinical Assessment

Caregivers completed a questionnaire, which included early life events, family demographics, past medical history, and current and previous medication use. Asthma symptom control was assessed using the Test for Respiratory and Asthma Control in Kids (TRACT) (Murphy et al., 2009) and pediatric asthma control test (PACT) (Zorc et al., 2006). Medical records regarding peak flow rate, pulmonary function test, and fractional exhaled nitric oxide (FeNO) were reviewed at our hospital where patients were treated. Blood was collected for differential leukocyte counts, total IgE, and specific IgE to aeroallergens (house dust mite, cat, dog, and grass pollen). Based on the levels of total IgE, patients were stratified into three classes: low (<200 I.U.), medium (200–1,000 I.U.), and high (>1,000 I.U.). For this study, peripheral blood mononuclear cells of subjects were also collected for typing genetic polymorphism of blood groups.

### Sample Collection and Preparation for 16S rRNA Sequencing

We collected microbiota samples using sterile cotton swabs from anterior nares of nasal cavities, retropharyngeal space, and rectum by anal insertions of 56 asthmatic children both during AE and in the RP. Swabbed samples were kept in 1.5 ml sterile saline buffer. All fresh samples were stored and transferred under optimal conditions, and were pretreated, i.e., being vortexed and centrifuged. DNA of airway and stool samples was extracted using QIAampR DNA Microbiome Kit and PowerFecal DNA Kit (*QIAGEN*, Hilden, Germany), respectively. Samples of low DNA quantity (<0.1 ng/μl) were excluded from sequencing. V3–V4 region of 16S rRNA gene was amplified using previously described primers (Klindworth et al., 2013). Amplicon samples were labeled dual-index barcodes using Nextera XT Index kit version 2 (Illumina), and libraries were prepared according to the Illumina MiSeq 16S Metagenomic Sequencing Protocol (Illumina, San Diego, Calif). Barcoded libraries were pooled for four runs of Illumina MiSeq 2 × 300 bp sequencing. AE and RP samples of the same individuals were sequenced in the same run.

### Data Preprocessing, Zero-Radius Operational Taxonomy Unit Clustering, and Taxonomy Annotation

Raw paired-end reads were merged using FLASH (v1.2.11) (Magoc and Salzberg, 2011), and short (<400 bp) merged reads were discarded. The preprocessed reads of all samples were clustered into zero-radius operation taxonomy units (ZOTUs) using UNOISE3 (in USEARCH v11.0.667) (Edgar, 2010, 2016). ZOTU sequences were annotated by RDP classifier (v2.12) (Wang et al., 2007), which was retrained to include species information. Unclassified sequences (score < 0.8 at the phylum level) were aligned to NCBI non-redundant database using MegaBLAST (Morgulis et al., 2008; Sayers et al., 2021), and those without the keyword 16S in the hits were removed from the ZOTU table, and samples with < 50,000 ZOTU reads were filtered. The resulting ZOTU table was also converted into taxon abundance table at different levels *via* the annotations by RDP classifier (confidence score cutoff 0.8). Percentages of the top 40 abundant taxa were visualized in a heatmap using the python package seaborn (v0.9). Specifically, clustermap (seaborn v0.9) was used to display hierarchical clustering with the average linkage method and braycurtis dissimilarity as the metric. Alpha and beta diversities were calculated using QIIME (v1.9) (Caporaso et al., 2010). Before analysis, the ZOTU table was rarified to the lowest total read count. Several alpha diversity indices (e.g., number of observed OTUs, ACE, Chao1, Shannon, and Simpson) were then calculated. In the beta diversity analysis, UniFrac distances were applied (Lozupone and Knight, 2005).

### Statistical Analyses

Correlation across classes of clinical features, as well as between microbial clustering and clinical features, were quantified by Fisher’s exact test in R (v3.6.2) (R Core Team, 2013). Association between a clinical feature and AE frequency was quantified by Kruskal-Wallis test in the SciPy package (v1.4.1). To compare microbial communities between groups of samples, we applied permutational multivariate ANOVA (PERMANOVA) in the vegan package (v2.5) of R using the UniFrac distances. Batch information was included in the regression models (i.e., distance ∼ batch + factor) to account for batch effect. Batch information was included in the adonis2 regression model (i.e., distance ∼ batch + factor; permutations = 9,999) to account for batch effect. ANCOM (v2.1) (Kaul et al., 2017) was used to identify differentially abundant taxons (feature_table_pre_process options: out_cut = 0.05, zero_cut = 0.9, lib_cut = 1,000, neg_lb = FALSE; ANCOM options: p_adjust_method = “BH,” alpha = 0.05, adj_formula = “batch,” rand_formula = “subject”), and all comparisons were adjusted for batch effect. A taxon was considered as differentially abundant if the *W*-value was ≥ 0.7. For paired analysis, linear mixed-effects model was applied with patient ID as the random intercept.

## Results

### Patient Characteristics

Clinical features (i.e., age, gender, IgE level and class, dust mite allergy, blood group, secretor status, Lewis type, pet-in-house, and subsequent AE frequency in a year) of 56 asthmatic children and adolescents were provided in Supplementary Table 1. Correlations between the clinical features were provided in Supplementary Table 2.

### Airway and Gut Microbiota

**FIGURE 1:**
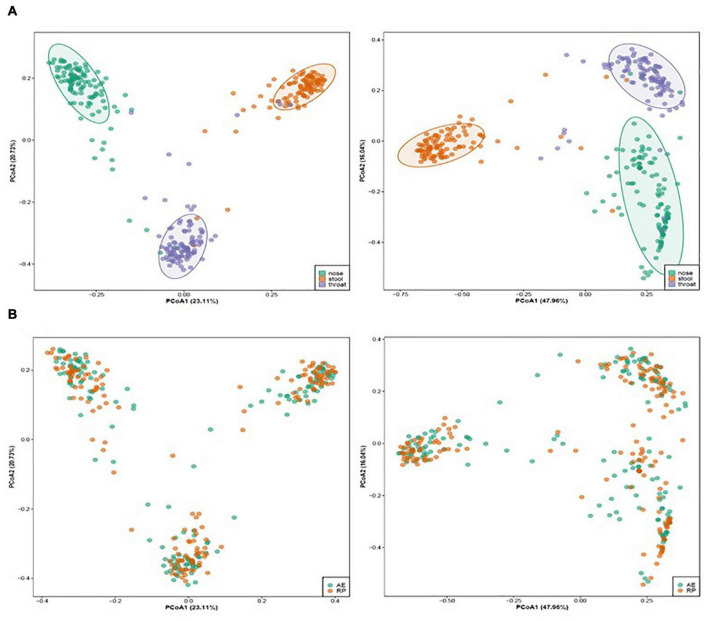
Un-weighted (left) and weighted (right) principal coordinate analysis of microbiota of asthmatic children colored by **(A)** body site (nose: green, throat: purple, stool: orange) and **(B)** asthmatic status (AE, green; RP, orange).

Totally, we collected paired, during AE and in the RP, nasal samples from 56 asthmatic patients, as well as 54 and 50 paired throat and stool samples, respectively, from these patients. For the 320 samples, MiSeq sequencing generated 45,587,776 raw paired-end reads, among which 39,732,495 could be merged and were long enough for community analysis. All processed reads were clustered into 58,425 ZOTUs. Two throat and one stool samples had < 50,000 ZOTU reads and were removed together with the corresponding AE or RP samples. This resulted in paired samples of 56, 52, and 49 individuals from the nose, throat, and stool sites, respectively. Beta diversity analysis on the ZOTUs revealed a clear separation of samples by body site except for few outliers (Figure 1A). Within each site, asthma status of AE and RP did not separate the samples (Figure 1B). Therefore, asthma status did not govern the overall structures of microbial communities.

### Alteration of Nasal Microbiota

**FIGURE 2:**
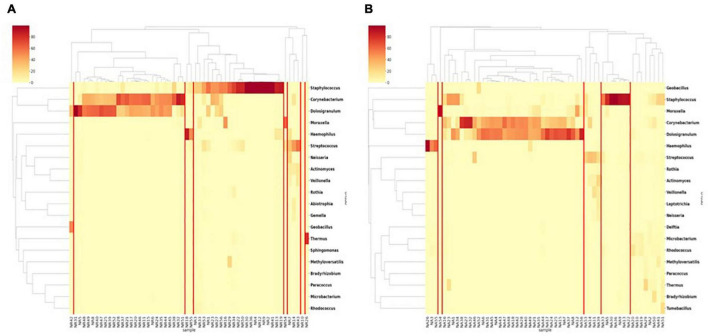
Clustering of nasal samples **(A)** in the RP and **(B)** during AE based on genus level composition.

In the RP samples as a baseline, nasal microbiota appeared in eight clusters (Figure 2A). In each cluster except one, the communities were dominated by one or two genera and the cluster was named as such. The two major clusters were *Corynecbacterium* + *Dolosigranulum* and *Staphylococcus*, which had 26 and 21 samples, respectively. During AE, the same five clusters appeared (Figure 2B), but with a different composition. Besides, a larger “mixed” cluster without a clear dominating genus was observed. The dominance in the *Streptococcus* cluster was also weaker than RP. For the two clusters, diversities of the AE samples were significantly higher relative to the matched RP samples (Supplementary Table 3). This suggests the nasal microbiota were more unstable during AE.

In both AE and RP samples, the *Streptococcus*, *Haemophilus*, and *Moraxella* clusters were relatively small. Among the six dominating genera, *Dolosigranulum*, *Haemophilus*, and *Moraxella* were majorly constituted by one single species, which were *D. pigrum*, *H. aegyptius*, and *M. catarrhalis*, respectively (Supplementary Figure 1). In the *Staphylococcus* dominated samples, two major species *S. aureus* and *S. capitis* were observed. Species compositions in the *Streptococcus* dominated samples were more diverse.

**FIGURE 3:**
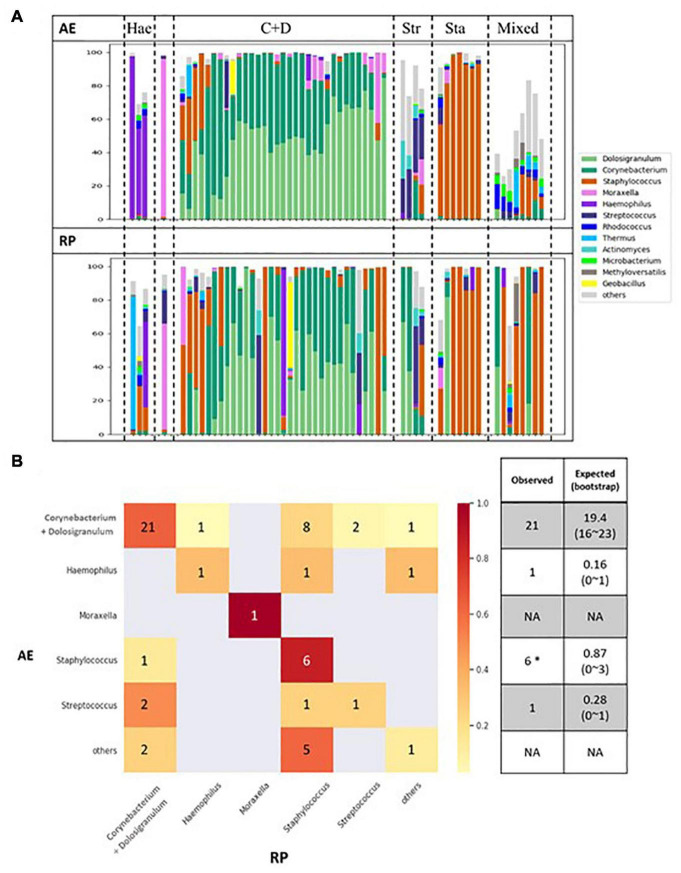
**(A)** Changes of microbial compositions in nasal samples moving from AE (top) into RP (bottom). **(B)** Transitions between microbial clusters from AE into RP. Expected number of self-transitions and the 95% interval are calculated assuming random transitions as described by Teo et al. (2015).

Figure 3 shows changes in microbial composition for all patients arranged based on the AE clusters. When moving into RP, the *Corynecbacterium* + *Dolosigranulum* cluster shrank and majority of the altered dominating genus switched to *Staphylococcus*. In contrast, six of the seven *Staphylococcus* dominating samples remained in the same cluster. Based on the weighted distances between paired samples, nasal microbiota remained relatively constant in 34 of the 56 patients (Supplementary Figure 2). Fisher’s exact test revealed that young children (age 3–5) tended to show a large change in nasal microbiota between AE and RP (*p* = 0.015) (Table 1); the calculated odds ratio (OR) was 4.83. Pairwise comparison of AE and RP samples revealed that *Staphylococcus* increased, while *Moraxella* and *Acinetobacter* decreased in relative abundance when moving into RP (Supplementary Figure 3). At the species level, the increments of both *Staphylococcus* species were also significant.

**TABLE 1:** Significance of association between clinical features and microbial clustering or microbiota for nasal samples.

Clinical feature	Microbial clustering during AE	Microbiota during AE	Microbial clustering in RP	Microbiota in RP	Change in microbiota
Age	**0.02646**	0.0875	0.486	0.1012	**0.0153**
Gender	0.4824	0.5684	0.8109	0.1599	0.5727
IgE class	**0.0206**	**0.0476**	0.3413	0.57	0.3779
Dust mite allergy	**0.0046**	**0.0212**	0.8109	0.789	0.549
Blood group	0.958	0.9185	0.9907	0.135	0.2287
Secretor status	0.07675	**0.0229**	0.4164	0.0629	0.2222
Lewis type	0.1365	**0.0174**	0.1176	0.0532	0.8115
Pet	0.588	0.973	0.2336	0.3193	0.7592
Subsequent AEs	0.1266	N.A.	0.7418	N.A.	0.4941

*Significant associations are in bold.*

*AE, acute exacerbation; RP, recovery phase.*

### Association Between Nasal Microbiota and Clinical Features

**FIGURE 4:**
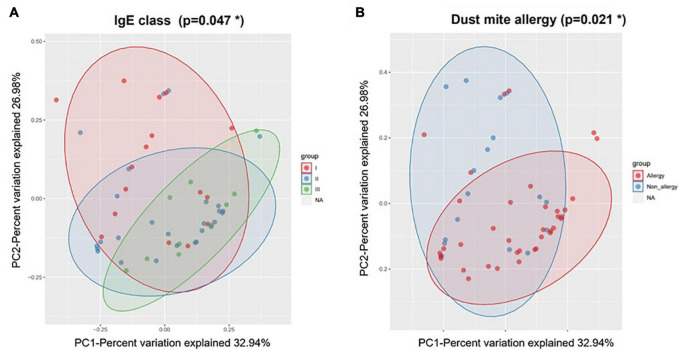
Weighted beta diversity of nasal samples during AE grouped by **(A)** IgE class and **(B)** dust mite allergy. *p < 0.05.

During AE, the microbial clustering was significantly associated with age, IgE class, and dust mite allergy (Table 1). Young children were nearly enriched in the *Streptococcus* cluster (OR = 9.75; *p* = 0.057), which diversity was higher, while depleted in the *Corynecbacterium* + *Dolosigranulum* cluster (OR = 0.21; *p* = 0.028) (Supplementary Table 4). Note that as age also correlated with allergy and IgE level (Supplementary Table 2), allergy might partially contribute to the association between age and microbial clustering. Allergic children were enriched in the *Corynecbacterium* + *Dolosigranulum* cluster (OR = 7.2; *p* = 0.002), and the *Corynecbacterium* + *Dolosigranulum* cluster had more children with a high IgE level (OR = 15; *p* = 0.014), which is reasonable as IgE levels in allergic children were higher (Supplementary Tables 1, 2). Consistently, nasal microbiota during AE were significantly different in different IgE classes and allergic reaction based on PERMANOVA (Table 1 and Figure 4). PERMANOVA also revealed a significant association between nasal microbiota and secretor status (*p* = 0.023) or Lewis type (*p* = 0.017). We repeated the above analyses for nasal samples in the RP and found no association between microbiota and all clinical features except the nearly significant association with secretor status or Lewis type (Table 1 and Supplementary Table 5). Therefore, our nasal microbiota were associated with certain clinical features only during AE.

### Throat and Stool Microbiota

**FIGURE 5:**
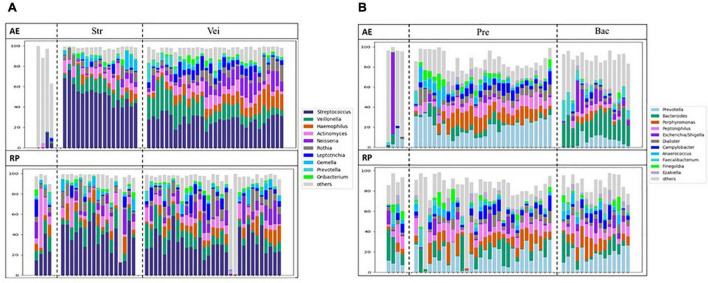
Changes of microbial compositions in **(A)** throat and **(B)** stool samples moving from AE (top) into RP (bottom).

Compared to nasal microbiota, throat microbiota were more complex and *Streptococcus* was usually the major genus (Supplementary Figure 4). The throat samples could be classified into three clusters, two of which showed different degrees of *Streptococcus* abundance, and the third one was excluded from the following analyses. During AE and in the RP, the microbial clustering was not associated with most clinical features (Supplementary Tables 6–8). The few significant associations were less convincing as the related analyses were not significant. For example, although throat microbiota was associated with dust mite allergy in the RP samples, the microbial clustering was not. In terms of changes in microbial compositions, no particular trend was observed (Figure 5A). Consistently, no genus showed a differential abundance between AE and RP of the same patients (Supplementary Figure 5). Thus, throat microbiota was not associated with asthma.

Except for few outliers, stool samples could be assorted into two main clusters (Supplementary Figure 6), which were represented by *Prevotella* and *Bacteroides*, respectively. When moving from AE into RP, the *Prevotella* cluster expanded, while the *Bacteroides* cluster shrank. During AE, the microbial clustering was not associated with any clinical feature except blood type (Supplementary Tables 9–11). Patients with A and AB type were enriched in the *Bacteroides* cluster, while those with B and O type tended to be in the *Prevotella* cluster (OR 11.67; *p* = 0.006). The association disappeared in the RP samples. In the RP, patients in the *Bacteroides* cluster showed more subsequent AEs compared to the *Prevotella* cluster (Supplementary Figure 7). The changes in microbial composition did not reveal a particular trend (Figure 5B). No other significant association or differentially abundant genus was found (Supplementary Table 9 and Supplementary Figure 8).

## Discussion

Currently, only few groups have inspected nasal microbiota in the same asthmatic children during AE and RP, respectively. Zhou et al. (2019) studied nasal microbiota in asthmatics at age 5–11 before and during an early loss of asthma control (yellow zone). In contrast, we investigated microbiota during and after AE. McCauley et al. (2019) examined nasal microbiota in asthmatics at age 6–17 every other week for 3 months, and additional nasal samples were collected during AE. In both studies, asthmatics in the U.S. were recruited. In contrast, we recruited asthmatics in Taiwan, a cohort with a different genetic background and living environment. Our simultaneous exploration of throat and stool microbiota also revealed the uniqueness of nasal microbiota. Therefore, this exploratory work provides a basis for studying airway and gut microbiota in Asian children and adolescents with asthma.

The clear separation of microbiota by body site is consistent with the current knowledge that body site is a major factor that shapes the microbial community. Although asthma status did not further separate the overall communities at each site, nasal microbiota during AE appeared special as only at that site and during that window of time did we find significant and consistent associations with clinical features, as well as distinct community structures. For example, more nasal sample showed a higher diversity during AE compared to RP. Similar was found when comparing yellow zone to the asymptotic state beforehand (Zhou et al., 2019). The high diversity suggests an unstable state and the unstable microbiota during AE might reflect interactions between the microbes and the agitated immunity. It is also reasonable that young children tended to show a larger change in nasal microbiota when moving from AE into RP as their immune system and microbiota were still evolving (Simon et al., 2015; Teo et al., 2018), and might be perturbed more during AE. For example, Teo et al. (2018) showed that the diversity of nasal microbiota started to increase from age 2 to 5 years.

Our nasal microbiota was associated with dust mite allergy and IgE class of the asthmatics only during AE. Interestingly, Teo et al. (2015) reported an association between *Haemophilus influenza*-specific IgG antibodies at 12 months of age and number of prior nasal samples with *Haemophilus* colonization only during acute respiratory illness (ARI). That is another example suggesting that nasal bacteria actively interact with the immune system only when the illness aggravates. During AE, the nasal microbiota of allergic children tended to be dominated by *Corynecbacterium* and *Dolosigranulum*. Nasal colonization of the two genera is often considered protective against AE. When under control, asthmatic children whose nasal microbiota was dominated by the two genera were less prone to progress into yellow zone (Zhou et al., 2019). Similarly, *Corynebacterium* was depleted in the noses of asthmatic children who later experienced AE (McCauley et al., 2019). These beneficial associations, however, were obtained based on the asymptomatic samples. In a study of nasal microbiota during ARI, *Corynebacterium* and *Dolosigranulum* were negatively associated with ARI in children before age 2 years (Teo et al., 2018). The association waned afterward and even became positive for *Dolosigranulum*. Therefore, the role of *Corynebacterium* and *Dolosigranulum* in the noses of asthmatic children during ARI was age dependent and could be positive.

The six dominating genera, *Corynebacterium*, *Dolosigranulum*, *Staphylococcus*, *Streptococcus*, *Haemophilus*, and *Moraxella*, have been reported in infants before age 1 year (Teo et al., 2015), in children through their first 5 years of life (Teo et al., 2018), and in older children and adolescents (Pérez-Losada et al., 2018; McCauley et al., 2019; Zhou et al., 2019). However, their compositions were different from ours. *Moraxella* was often a prevalent dominating genus in nasal microbiota (Depner et al., 2017; Teo et al., 2018; McCauley et al., 2019), e.g., in 40% of nasal samples of children in their first 5 years of life (Teo et al., 2018). In contrast, the domination was found in only one of our 56 nasal samples during AE or in the RP. The distinction could be partly explained by age. As nasal microbiota in adults are usually lacking of *Moraxella*, *Streptococcus*, and *Haemophilus* (Kumpitsch et al., 2019), we observed that all the samples dominated by *Moraxella*, *Streptococcus*, and *Haemophilus* were from patients under age 11. Geographic factor may also play a role. In the studies of McCauley et al. (2019) and Zhou et al. (2019), *Moraxella* was dominating in 34 and 6% of the cohorts, respectively. Despite our only one *Moraxella* dominating sample, *Moraxella* did exist in several AE samples and the percentage decreased significantly when moving into RP, consistent with its pathogenic role in asthma (Alnahas et al., 2017; Teo et al., 2018).

When moving into RP, the relative abundance of *Staphylococcus* increased. The role of *Staphylococcus* in asthma development is still controversial. Zhou et al. (2019) found that *Staphylococcus* was associated with progression into yellow zone. However, McCauley et al. (2019) showed that the *Staphylococcus* dominated nasal microbiota was associated with reduced ARI and exacerbation events. Species level investigation may reconcile the controversy. Zhou et al. (2019) probed the V1–V3 regions and found that *S. aureus* was the major species. We probed the V3–V4 regions and identified *S. aureus* and *S. capitis* (close to *S. epidermidis*) (George and Kloos, 1994) as the two major species. *S. aureus* colonization was found associated with wheeze and asthma in the U.S. children and adolescents (Davis et al., 2015), and likely orchestrated severe airway inflammation (Bachert et al., 2020). In contrast, isolates of *S. epidermidis* have been shown to stimulate nasal epithelia to produce antimicrobial peptides, contributing to the healthy maturation of nasal microbiota (Liu et al., 2020). The opposite role of the two species is also supported by the finding that *S. aureus*, but not *S. epidermidis*, increased the epithelial damage (Lan et al., 2018; McCauley et al., 2019). The roles of *Staphylococcus* species in childhood asthma thus need further investigation.

Our nasal microbiota was associated with secretor status during AE. The association was also borderline significant in the RP samples. This suggests a host genetic factor modulating nasal microbiota. In the stool samples during AE, we also found that patients with A or AB type were enriched in the *Bacteroides* cluster. Interestingly, a significant difference in gut microbiota between individuals of blood group A and others has been reported (Gampa et al., 2017). Our finding suggests the importance of interactions between host glycobiology and gut microbiota in asthma, which deserves future investigation.

Environmental and host factors both affect the throat and nasal microbiota. Wagner Mackenzie et al. (2019) showed that seasonal changes and individual differences could explain majority of variation in nasal microbiota. The seasonal factors, however, played a smaller role compared to host factors (∼10% vs. ∼50% of the variation). Moreover, nasal microbiota of the same individual remained relatively stable within the same season (Wagner Mackenzie et al., 2019). As our samples during asthma exacerbation and in the recovery phase were taken only 2 weeks apart, we expect that the environmental factors remained relatively constant and did not affect much the microbiota. In addition, for identifying differentially abundant microbes, we compared samples of the same individuals to eliminate the concern of individual differences. In another study investigating the relationship between greenness and nasal microbiome, no association was found (Gisler et al., 2006).

Our stool microbiota in RP was associated with the frequency of subsequent AEs. The association between gut microbiota and asthma in older children and adolescents is much less studied because the early life window is known critical for development of immune system and asthma (Stokholm et al., 2018). Our discovered association between asymptotic stool microbiota and frequency of subsequent AEs suggests that gut microbes in older children and adolescents could also impact the subsequent exacerbation.

## Conclusion

The fact that our throat and stool microbiota were not associated with most clinical features of asthma indicates the uniqueness of nasal cavity in childhood asthma. Previous works examining upper airway microbes in asthma also reveal that alterations in nasal microbiota, but not throat microbiota, were associated with asthma (Boutin et al., 2017; Depner et al., 2017). In this work, we further point out that AE was a specific window of time for nasal microbes to interact with the local immunity.

## Data Availability Statement

The datasets presented in this study can be found in online repositories. The names of the repository/repositories and accession number(s) can be found below: https://www.ncbi.nlm.nih.gov/, PRJNA662456.

## Ethics Statement

This study protocol was approved by the Ethical and Clinical Trial Committee and the investigation review board of National Cheng Kung University Hospital (A-BR106-069). All patients’ caregivers provided written informed consent. Written informed consent to participate in this study was provided by the participants’ legal guardian/next of kin.

## Author Contributions

TL, LW, and J-YW conceived the research and designed the experiments. J-YW, TL, H-JT, and LW wrote the manuscript. W-SK, M-HH, and P-CC collected samples and clinical information from the asthmatic patients. Y-LC and C-LH performed the experiments of DNA extraction and 16S rRNA gene amplification. LW did the initial data analysis. TL and C-HL analyzed all the data and generated figures and tables. S-LJ ensured validity of the statistical analyses. All authors have read and approved the manuscript.

## Conflict of Interest

The authors declare that the research was conducted in the absence of any commercial or financial relationships that could be construed as a potential conflict of interest.

## Publisher’s Note

All claims expressed in this article are solely those of the authors and do not necessarily represent those of their affiliated organizations, or those of the publisher, the editors and the reviewers. Any product that may be evaluated in this article, or claim that may be made by its manufacturer, is not guaranteed or endorsed by the publisher.

## Abbreviations

AE: acute exacerbation
RP: recovery phase
IgE: immunoglobulin E
ARI: acute respiratory illness
ZOTU: zero-radius operational taxonomy unit
PERMANOVA: permutational multivariate ANOVA
OR: odds ratio.
