# Supplementary material for: Nasal Microbiome Change During and After Exacerbation in Asthmatic Children
Source: Front Microbiol. 2022 Mar 4;12:833726. doi: 10.3389/fmicb.2021.833726 (PMC8931732; doi:10.3389/fmicb.2021.833726)
Supplement: Supplementary file 1 [file Data_Sheet_1.docx]

**Supplementary Materials**

**Liu *et al.*** Nasal Microbiome Change During and After Exacerbation in Asthmatic Children

**Supplementary Tables**

**Table S1**. Patient characteristics.

| Patient | Age | Gender | IgE level | IgE class | Dust mite allergy | Blood type | Secretor status | Lewis type | Pet | SAE | Note |
| --- | --- | --- | --- | --- | --- | --- | --- | --- | --- | --- | --- |
| P1 | 12 | F | 274 | II | Yes | O | Se/Se | a-b+ | No | 4 |  |
| P2 | 7 | M | 77.2 | I | No | O | Se^w^/Se^w^ | a+b+ | No | 1 |  |
| P3 | 6 | F | 45.5 | I | No | A | Se/Se^w^ | a-b+ | No | 1 |  |
| P4 | 8 | M | 1049 | III | Yes | NA | NA | NA | Yes | 1 |  |
| P5 | 5 | F | NA | NA | No | O | Se/Se^w^ | a-b+ | No | 4 |  |
| P6 | 10 | M | 723 | II | Yes | O | Se/Se^w^ | a-b+ | No | 1 |  |
| P7 | 6 | M | 253 | II | Yes | O | Se/Se | a-b+ | No | 1 |  |
| P8 | 7 | M | 188 | I | No | B | Se/Se | a-b+ | No | 2 | No stool |
| P9 | 8 | M | 241 | II | Yes | O | Se/Se^w^ | a-b+ | Yes | 1 |  |
| P10 | 7 | M | 281 | II | Yes | B | Se/Se | a-b+ | Yes | 1 |  |
| P11 | 5 | M | 167 | I | No | A | Se/Se | a-b+ | No | 2 | No stool |
| P12 | 6 | M | 665 | II | Yes | A | Se/Se^w^ | a-b+ | No | 1 |  |
| P13 | 6 | M | 925 | II | Yes | B | Se/Se^w^ | a-b+ | Yes | 1 |  |
| P14 | 10 | M | 1351 | III | Yes | B | Se/Se^w^ | a-b+ | No | 1 |  |
| P15 | 4 | F | 863 | II | Yes | AB | Se/Se^w^ | a-b+ | Yes | 1 |  |
| P16 | 5 | M | 1014 | III | Yes | NA | NA | NA | No | 3 |  |
| P17 | 6 | M | 2574 | III | Yes | O | Se/Se^w^ | a-b+ | No | 5 |  |
| P18 | 6 | M | 1434 | III | Yes | NA | NA | NA | Yes | 1 |  |
| P19 | 5 | M | 52.2 | I | No | O | Se/Se^w^ | a-b+ | No | 4 |  |
| P20 | 9 | M | 956 | II | Yes | B | Se/Se | a-b+ | No | 1 |  |
| P21 | 6 | M | 101 | I | No | NA | NA | NA | No | 2 |  |
| P22 | 13 | M | NA | NA | No | NA | NA | NA | No | 1 |  |
| P23 | 13 | F | 226 | II | Yes | AB | Se/Se^w^ | a+b- | Yes | 4 |  |
| P24 | 17 | M | 4076 | III | Yes | AB | Se/Se^w^ | a+b- | Yes | 1 |  |
| P25 | 6 | M | 104 | I | No | B | Se/Se^w^ | a-b+ | No | 1 |  |
| P26 | 3 | M | 110.0 | I | NA | B | Se^w^/Se^w^ | a+b+ | No | 3 |  |
| P27 | 6 | F | 1327 | III | Yes | A | Se/Se^w^ | a-b+ | No | 1 |  |
| P28 | 10 | F | 587 | II | Yes | B | Se/Se^w^ | a-b+ | No | 2 |  |
| P29 | 6 | F | 265 | II | Yes | B | Se/Se | a-b+ | No | 1 | No stool & throat |
| P30 | 8 | M | 996 | II | Yes | O | Se^w^/Se^w^ | a+b+ | No | 2 |  |
| P31 | 4 | M | 340 | II | Yes | O | Se/Se | a-b+ | No | 1 |  |
| P32 | 6 | F | 264 | II | Yes | NA | NA | NA | No | 1 |  |
| P33 | 5 | M | 78.9 | I | No | A | Se^w^/Se^w^ | a+b+ | Yes | 2 |  |
| P34 | 11 | M | 1046 | III | Yes | NA | NA | NA | No | 1 |  |
| P35 | 6 | F | 823 | II | Yes | B | Se/Se | a-b+ | No | 5 |  |
| P36 | 11 | M | 497 | II | Yes | NA | NA | NA | No | 1 |  |
| P37 | 11 | F | 618 | II | Yes | NA | NA | NA | No | 1 |  |
| P38 | 17 | M | 95.0 | I | NA | A | Se^w^/Se^w^ | a-b- | No | 0 |  |
| P39 | 5 | M | 86.7 | I | No | O | Se/Se | a-b+ | No | 3 |  |
| P40 | 9 | M | 376 | II | Yes | NA | NA | NA | No | 1 |  |
| P41 | 4 | F | 152 | I | No | NA | NA | NA | No | 3 |  |
| P42 | 5 | M | 385 | II | Yes | B | Se/Se^w^ | a-b+ | Yes | 2 | No stool |
| P43 | 3 | F | 15.4 | I | No | NA | NA | NA | No | 4 | No stool |
| P44 | 9 | F | 245 | II | Yes | O | Se/Se | a-b+ | No | 3 |  |
| P45 | 10 | M | 257 | II | Yes | O | Se/Se^w^ | a-b+ | No | 1 |  |
| P46 | 6 | F | 779 | II | Yes | O | Se/Se^w^ | a-b+ | Yes | 1 |  |
| P47 | 6 | M | 2529 | III | Yes | O | Se/Se^w^ | a-b+ | No | 5 |  |
| P48 | 6 | F | 407 | II | Yes | A | Se/Se^w^ | a-b+ | Yes | 2 |  |
| P49 | 6 | F | 56.2 | I | No | AB | Se/Se | a-b+ | No | 1 |  |
| P50 | 5 | M | 43.6 | I | No | O | Se/Se | a-b+ | Yes | 1 |  |
| P51 | 11 | M | 368 | II | Yes | A | Se/Se^w^ | a-b+ | No | 1 |  |
| P52 | 7 | M | 912 | II | Yes | NA | NA | NA | No | 2 | No stool & throat |
| P53 | 5 | M | 61.5 | I | No | O | Se^w^/Se^w^ | a+b+ | No | 5 |  |
| P54 | 6 | F | 593 | II | Yes | B | Se^w^/Se^w^ | a+b+ | Yes | 1 |  |
| P55 | 4 | M | NA | NA | No | NA | NA | NA | No | 1 |  |
| P56 | 9 | M | 1906 | III | Yes | B | Se/Se | a-b+ | Yes | 3 |  |

Note:

IgE class (I: <200; II: 200-1000; III: >1000). SAE: number of subsequent acute exacerbation events in a year.

**Table S2**. P-values of correlations between clinical features.

|  | Age | Gender | IgE class | Dust mite allergy | Blood group | Secretion type | Lewis type | Pet | AE |
| --- | --- | --- | --- | --- | --- | --- | --- | --- | --- |
| Age |  |  |  |  |  |  |  |  |  |
| Gender | 0.7508 |  |  |  |  |  |  |  |  |
| IgE class | 0.0033 | 0.1265 |  |  |  |  |  |  |  |
| Dust mite allergy | 0.00042 | 0.7635 | 7.7e-13 |  |  |  |  |  |  |
| Blood group | 0.7329 | 0.2771 | 0.9105 | 0.641 |  |  |  |  |  |
| Secretor status | 0.4072 | 0.6129 | 0.0715 | 0.1634 | 0.763 |  |  |  |  |
| Lewis type | 0.2943 | 0.7895 | 0.1474 | 0.2881 | 0.07115 | 3.01e-8 |  |  |  |
| Pet | 1 | 1 | 0.203 | 0.1056 | 0.1508 | 0.6765 | 0.2232 |  |  |
| SAE | 0.00631 | 0.2664 | 0.2917 | 0.14039 | 0.3359 | 0.8839 | 0.2473 | 0.2892 |  |

Note:

Significant associations are indicated in red.

**Table S3**. Pairwise comparison of Shannon diversity for nasal samples in the AE clusters.

| Cluster | Average diversity during AE | Average diversity in recovery | p-value |
| --- | --- | --- | --- |
| Haemophilus | 1.112 | 2.26 | 0.108 |
| Corynebacterium+Dolosigranulum | 1.27 | 1.159 | 0.416 |
| Streptococcus | 3.082 | 1.84 | 0.067 |
| Staphylococcus | 0.711 | 0.833 | 0.398 |
| Mixed | 2.604 | 1.115 | 0.049 |
| Streptococcus+Mixed | 2.763 | 1.356 | 0.012 |
| All | 1.571 | 1.229 | 0.113 |

**Table S4a**. Distribution of nasal samples in the AE clusters and clinical features. (Hae=*Haemophilus*; C+D=*Corynebacterium* and *Dolosigranulum*; Str=*Streptococcus*; Sta=*Staphylococcus*)

| Gender (*p*=0.482)  Cluster | | | | | | |
| --- | --- | --- | --- | --- | --- | --- |
| Class | Hae | C+D | Str | Sta | Mixed | Sum |
| M | 2 | 20 | 4 | 6 | 6 | 38 |
| F | 1 | 13 | 0 | 1 | 2 | 17 |
| Sum | 3 | 33 | 4 | 7 | 8 | 55 |
| IgE class (*p*=0.0206 ) | | | | | | |
| I | 2 | 6 | 3 | 1 | 4 | 16 |
| II | 0 | 18 | 0 | 4 | 4 | 26 |
| III | 0 | 9 | 1 | 0 | 0 | 10 |
| Sum | 2 | 33 | 4 | 5 | 8 | 52 |
| Blood group (*p*=0.958) | | | | | | |
| A | 1 | 4 | 1 | 1 | 1 | 8 |
| B | 1 | 6 | 1 | 1 | 3 | 12 |
| AB | 0 | 4 | 0 | 0 | 0 | 4 |
| O | 0 | 9 | 2 | 3 | 3 | 17 |
| Sum | 2 | 23 | 4 | 5 | 7 | 41 |
| Secretor status (*p*=0.076) | | | | | | |
| Se/Se | 0 | 8 | 2 | 0 | 4 | 14 |
| Se/Se^w^ | 1 | 14 | 1 | 3 | 2 | 21 |
| Se^w^/Se^w^ | 1 | 1 | 1 | 2 | 1 | 6 |
| Sum | 2 | 23 | 4 | 5 | 7 | 41 |
| Lewis type (*p*=0.136) | | | | | | |
| a+b+ | 1 | 0 | 1 | 2 | 1 | 5 |
| a+b- | 0 | 2 | 0 | 0 | 0 | 2 |
| a-b+ | 1 | 20 | 3 | 3 | 6 | 33 |
| a-b- | 0 | 1 | 0 | 0 | 0 | 1 |
| Sum | 2 | 23 | 4 | 5 | 7 | 41 |
| Dust mite allergy (*p*=0.004) | | | | | | |
| Allergic | 0 | 27 | 1 | 4 | 4 | 36 |
| Non-allergic | 2 | 5 | 3 | 3 | 4 | 17 |
| Sun | 2 | 32 | 4 | 7 | 8 | 53 |
| Pet (*p*=0.588) | | | | | | |
| Yes | 0 | 9 | 0 | 3 | 2 | 14 |
| No | 3 | 24 | 4 | 4 | 6 | 41 |
| Age (*p*=0.0264) | | | | | | |
| 3~5 | 2 | 5 | 3 | 2 | 3 | 15 |
| 6~17 | 1 | 28 | 1 | 5 | 5 | 40 |
| Sum | 3 | 33 | 4 | 7 | 8 | 55 |

**Table S4b**. Distribution of nasal samples in the AE clusters and clinical features in a 2x2 format.

| IgE class (OR=0.067; *p*=0.014)  Cluster | | | |
| --- | --- | --- | --- |
| Class | C+D | Non C+D | Sum |
| Low | 6 | 10 | 16 |
| High | 9 | 1 | 10 |
| Sum | 15 | 11 | 26 |
| Dust mite allergy (OR=7.2; *p*=0.002)  Cluster | | | |
| Class | C+D | Non C+D | Sum |
| Allergic | 27 | 9 | 36 |
| Non-allergic | 5 | 12 | 17 |
| Sum | 32 | 21 | 53 |
| Age (OR=9.75; p=0.057)  Cluster | | | |
| Class | Str | Non Str | Sum |
| 3~5 | 3 | 12 | 15 |
| 6~17 | 1 | 39 | 40 |
| Sum | 4 | 51 | 55 |
| Age (OR=0.214; p=0.028)  Cluster | | | |
| Class | C+D | Non C+D | Sum |
| 3~5 | 5 | 10 | 15 |
| 6~17 | 28 | 12 | 40 |
| Sum | 33 | 22 | 55 |

**Table S5**. Distribution of nasal samples in the RP clusters and clinical features.

| Gender (p=0.81) | | | | | |
| --- | --- | --- | --- | --- | --- |
| Cluster  Class | C+D | Hae | Sta | Str | Sum |
| M | 16 | 2 | 15 | 2 | 35 |
| F | 10 | 0 | 6 | 1 | 17 |
| Sum | 26 | 2 | 21 | 3 | 52 |
| IgE class (p=0.341) | | | | | |
| I | 7 | 0 | 7 | 1 | 15 |
| II | 15 | 0 | 8 | 1 | 24 |
| III | 3 | 1 | 5 | 1 | 10 |
| Sum | 25 | 1 | 20 | 3 | 49 |
| Blood group (p=0.99) | | | | | |
| A | 4 | 0 | 3 | 0 | 7 |
| B | 5 | 0 | 3 | 1 | 9 |
| AB | 3 | 0 | 1 | 0 | 4 |
| O | 9 | 0 | 7 | 1 | 17 |
| Sum | 21 | 0 | 14 | 2 | 37 |
| Secretor status (p=0.416) | | | | | |
| Se/Se | 8 | 0 | 4 | 1 | 13 |
| Se/Se^w^ | 12 | 0 | 7 | 1 | 20 |
| Se^w^/Se^w^ | 1 | 0 | 4 | 0 | 5 |
| Sum | 21 | 0 | 15 | 2 | 38 |
| Lewis type (p=0.117) | | | | | |
| a+b+ | 0 | 0 | 4 | 0 | 4 |
| a+b- | 1 | 0 | 1 | 0 | 2 |
| a-b+ | 19 | 0 | 10 | 2 | 31 |
| a-b- | 1 | 0 | 0 | 0 | 1 |
| Sum | 21 | 0 | 15 | 2 | 38 |
| Dust mite allergy (p=0.81) | | | | | |
| Allergic | 18 | 1 | 13 | 2 | 34 |
| Non-allergic | 7 | 1 | 8 | 1 | 17 |
| Sun | 25 | 2 | 21 | 3 | 51 |
| Pet (p=0.233) | | | | | |
| Yes | 4 | 1 | 7 | 0 | 12 |
| No | 22 | 1 | 14 | 3 | 40 |
| Sum | 26 | 2 | 21 | 3 | 52 |
| Age (p=0.486) | | | | | |
| 3~5 | 5 | 1 | 6 | 1 | 13 |
| 6~17 | 21 | 1 | 15 | 2 | 39 |
| Sum | 26 | 2 | 21 | 3 | 52 |

**Table S6**. Significance of association between clinical features and microbial clustering and microbiota for throat samples.

| Clinical feature | Microbial clustering during attack | Microbiota during attack | Microbial clustering in recovery | Microbiota in recovery |
| --- | --- | --- | --- | --- |
| Age | 0.485 | 0.86 | 0.736 | 0.985 |
| Gender | 0.547 | 0.019 | 1 | 0.289 |
| IgE class | 0.84 | 0.347 | 0.731 | 0.374 |
| Dust mite allergy | 1 | 0.699 | 0.196 | 0.027 |
| Blood group | 0.876 | 0.936 | 0.091 | 0.513 |
| Secretor status | 0.887 | 0.493 | 0.132 | 0.255 |
| Lewis type | 1 | 0.904 | 0.228 | 0.642 |
| Pet | 0.497 | 0.556 | 0.307 | 0.22 |
| AE | 0.677 | N.A. | 0.224 | N.A. |

Note:

Significant associations are in red.

**Table S7**. Distribution of throat samples in the AE clusters and clinical features. (Str=*Streptococcus*; Vei=*Veillonella*)

| Gender (p=0.547)  Cluster | | | |
| --- | --- | --- | --- |
| Class | Str | Vei | Sum |
| M | 10 | 21 | 31 |
| F | 7 | 10 | 17 |
| Sum | 17 | 31 | 48 |
| IgE class (p=0.84) | | | |
| I | 3 | 8 | 11 |
| II | 8 | 16 | 24 |
| III | 4 | 6 | 10 |
| Sum | 15 | 30 | 45 |
| Blood group (p=0.876) | | | |
| A | 2 | 3 | 5 |
| B | 5 | 7 | 12 |
| AB | 2 | 2 | 4 |
| O | 4 | 10 | 14 |
| Sum | 13 | 22 | 35 |
| Secretor status (p=0.887) | | | |
| Se/Se | 5 | 6 | 11 |
| Se/Se^w^ | 6 | 13 | 19 |
| Se^w^/Se^w^ | 2 | 3 | 5 |
| Sum | 13 | 22 | 35 |
| Lewis type (p=1) | | | |
| a+b+ | 2 | 3 | 5 |
| a+b- | 1 | 1 | 2 |
| a-b+ | 10 | 18 | 28 |
| a-b- | 0 | 0 | 0 |
| Sum | 13 | 22 | 35 |
| Dust mite allergy (p=1) | | | |
| Allergic | 12 | 22 | 34 |
| Non-allergic | 5 | 8 | 13 |
| Sun | 17 | 30 | 47 |
| Pet (p=0.497) | | | |
| Yes | 3 | 9 | 12 |
| No | 13 | 21 | 34 |
| Sum | 16 | 30 | 46 |
| Age (p=0.486) | | | |
| 3~5 | 5 | 6 | 11 |
| 6~17 | 12 | 25 | 37 |
| Sum | 17 | 31 | 48 |

**Table S8**. Distribution of throat samples in the RP clusters and clinical features. (Str=*Streptococcus*; Lep=*Leptotrichia*)

| Gender (p=1)  Cluster | | | |
| --- | --- | --- | --- |
| Class | Str | Lep | Sum |
| M | 13 | 19 | 32 |
| F | 7 | 9 | 16 |
| Sum | 20 | 28 | 48 |
| IgE class (p=0.731) | | | |
| I | 4 | 8 | 12 |
| II | 11 | 12 | 23 |
| III | 4 | 6 | 10 |
| Sum | 19 | 26 | 45 |
| Blood group (p=0.091) | | | |
| A | 6 | 1 | 7 |
| B | 4 | 8 | 12 |
| AB | 2 | 2 | 4 |
| O | 4 | 9 | 13 |
| Sum | 16 | 20 | 36 |
| Secretor status (p=0.132) | | | |
| I | 3 | 9 | 12 |
| II | 10 | 9 | 19 |
| III | 2 | 9 | 11 |
| Sum | 15 | 27 | 42 |
| Lewis type (p=0.228) | | | |
| a+b+ | 2 | 2 | 4 |
| a+b- | 2 | 0 | 2 |
| a-b+ | 11 | 18 | 29 |
| a-b- | 1 | 0 | 1 |
| Sum | 16 | 20 | 36 |
| Dust mite allergy (p=0.196) | | | |
| Allergic | 15 | 18 | 33 |
| Non-allergic | 3 | 10 | 13 |
| Sun | 18 | 28 | 46 |
| Pet (p=0.307) | | | |
| Yes | 3 | 9 | 12 |
| No | 16 | 18 | 34 |
| Sum | 19 | 27 | 46 |
| Age (p=0.736) | | | |
| 3~5 | 4 | 8 | 12 |
| 6~17 | 16 | 20 | 36 |
| Sum | 20 | 28 | 48 |

**Table S9**. Significance of association between clinical features and microbial clustering and microbiota for stool samples.

| Clinical feature | Microbial clustering during attack | Microbiota during attack | Microbial clustering in recovery | Microbiota in recovery | Change in microbiota |
| --- | --- | --- | --- | --- | --- |
| Age | 1 | 0.307 | 0.678 | 0.248 | 0.45 |
| Gender | 0.091 | 0.154 | 1 | 0.23 | 0.188 |
| IgE class | 0.701 | 0.402 | 0.787 | 0.611 | 0.545 |
| Dust mite allergy | 1 | 0.167 | 0.434 | 0.239 | 1 |
| Blood group | 0.026 | 0.148 | 1 | 0.678 | 0.418 |
| Secretor status | 0.289 | 0.673 | 0.839 | 0.763 | 0.857 |
| Lewis type | 0.822 | 0.849 | 1 | 0.904 | 0.774 |
| Pet | 1 | 0.601 | 1 | 0.812 | 1 |
| SAE | 0.488 | N.A. | 0.018 | N.A. |  |

Note:

Significant associations are in red.

**Table S10**. Distribution of stool samples in the AE clusters and clinical features. (Pre=*Prevotella*; Bac=*Bacteroides*)

| Gender (p=0.091)  Cluster | | | |
| --- | --- | --- | --- |
| Class | Pre | Bac | Sum |
| M | 23 | 7 | 30 |
| F | 7 | 8 | 15 |
| Sum | 30 | 15 | 45 |
| IgE class (p=0.701) | | | |
| I | 7 | 4 | 11 |
| II | 14 | 9 | 23 |
| III | 7 | 2 | 9 |
| Sum | 28 | 15 | 43 |
| Blood group (p=0.026) | | | |
| A | 1 | 4 | 5 |
| B | 8 | 1 | 9 |
| AB | 1 | 3 | 4 |
| O | 12 | 5 | 17 |
| Sum | 22 | 13 | 35 |
| Blood group (OR = 0.086; p=0.006) | | | |
| A & AB | 2 | 7 | 9 |
| B & O | 20 | 6 | 26 |
| Sum | 22 | 13 | 35 |
| Secretor status (p=0.289) | | | |
| Se/Se | 8 | 2 | 10 |
| Se/Se^w^ | 10 | 10 | 20 |
| Se^w^/Se^w^ | 4 | 1 | 5 |
| Sum | 22 | 13 | 35 |
| Lewis type (p=0.822) | | | |
| a+b+ | 4 | 1 | 5 |
| a+b- | 1 | 1 | 2 |
| a-b+ | 17 | 11 | 28 |
| a-b- | 0 | 0 | 0 |
| Sum | 22 | 13 | 35 |
| Dust mite allergy (p=1) | | | |
| Allergic | 21 | 11 | 32 |
| Non-allergic | 8 | 4 | 12 |
| Sun | 29 | 15 | 44 |
| Pet (p=1) | | | |
| Yes | 7 | 3 | 10 |
| No | 22 | 11 | 33 |
| Sum | 29 | 14 | 43 |
| Age (p=1) | | | |
| 3~5 | 7 | 3 | 10 |
| 6~17 | 23 | 12 | 35 |
| Sum | 30 | 15 | 45 |

**Table S11**. Distribution of stool samples in the RP clusters and clinical features. (Pre=*Prevotella*; Bac=*Bacteroides*)

| Gender (p=1)  Cluster | | | |
| --- | --- | --- | --- |
| Class | Pre | Bac | Sum |
| M | 24 | 7 | 31 |
| F | 13 | 3 | 16 |
| Sum | 37 | 10 | 47 |
| IgE class (p=0.787) | | | |
| I | 8 | 3 | 11 |
| II | 20 | 4 | 24 |
| III | 7 | 2 | 9 |
| Sum | 35 | 9 | 44 |
| Blood group (p=1) | | | |
| A | 6 | 1 | 7 |
| B | 7 | 2 | 9 |
| AB | 4 | 0 | 4 |
| O | 13 | 3 | 16 |
| Sum | 30 | 6 | 36 |
| Secretor status (p=0.839) | | | |
| Se/Se | 10 | 1 | 11 |
| Se/Se^w^ | 16 | 4 | 20 |
| Se^w^/Se^w^ | 4 | 1 | 5 |
| Sum | 30 | 6 | 36 |
| Lewis type (p=1) | | | |
| a+b+ | 3 | 1 | 4 |
| a+b- | 2 | 0 | 2 |
| a-b+ | 24 | 5 | 29 |
| a-b- | 1 | 0 | 1 |
| Sum | 30 | 6 | 36 |
| Dust mite allergy (p=0.434) | | | |
| Allergic | 27 | 6 | 33 |
| Non-allergic | 9 | 4 | 13 |
| Sun | 36 | 10 | 46 |
| Pet (p=1) | | | |
| Yes | 9 | 2 | 11 |
| No | 27 | 7 | 34 |
| Sum | 36 | 9 | 45 |
| Age (p=0.678) | | | |
| 3~5 | 8 | 3 | 11 |
| 6~17 | 29 | 7 | 36 |
| Sum | 37 | 10 | 47 |

**Supplementary Figures**


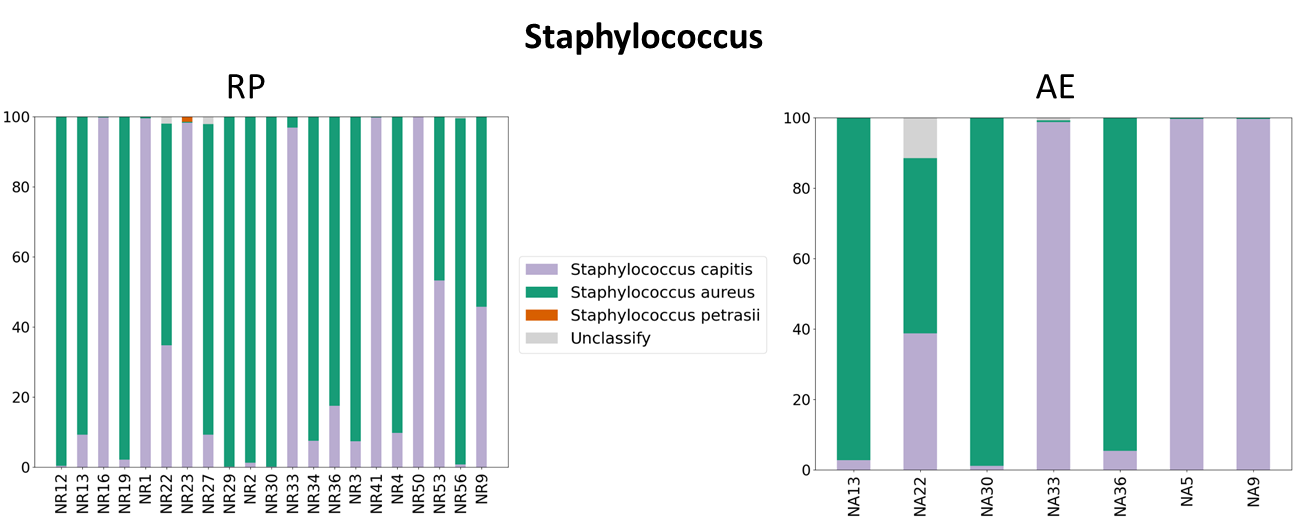


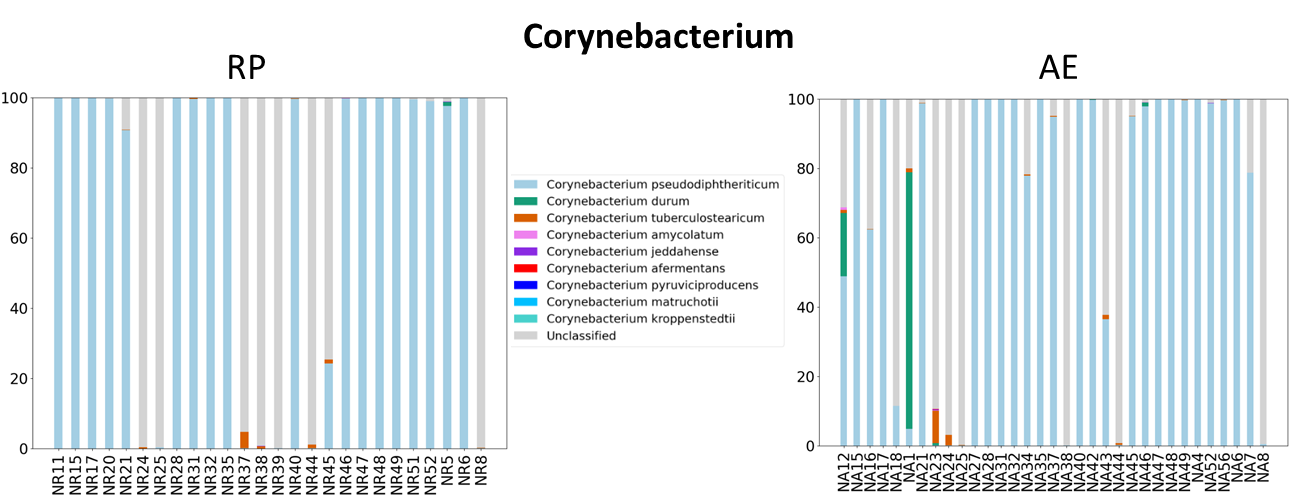


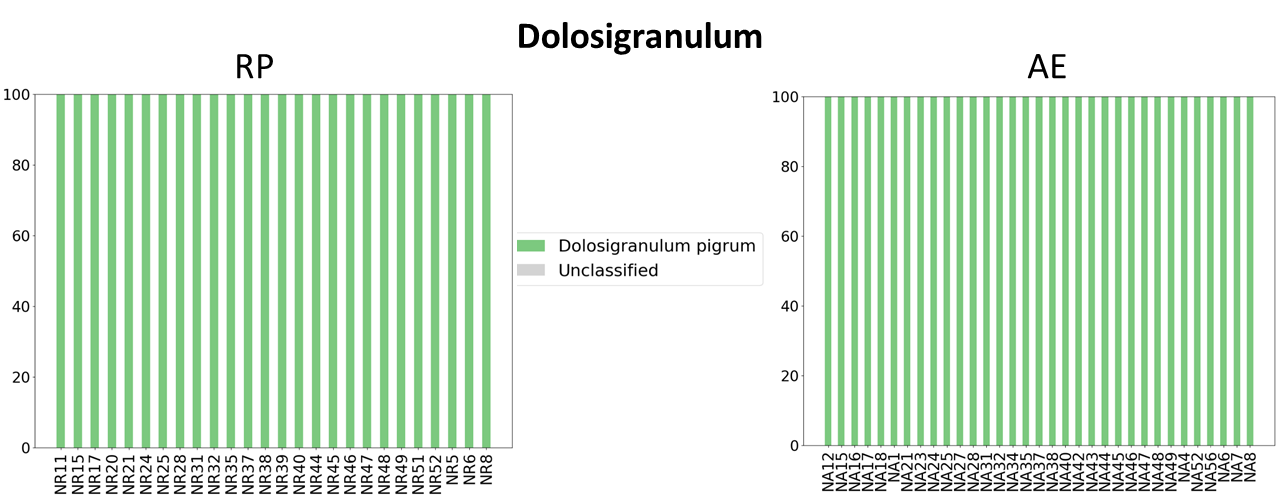


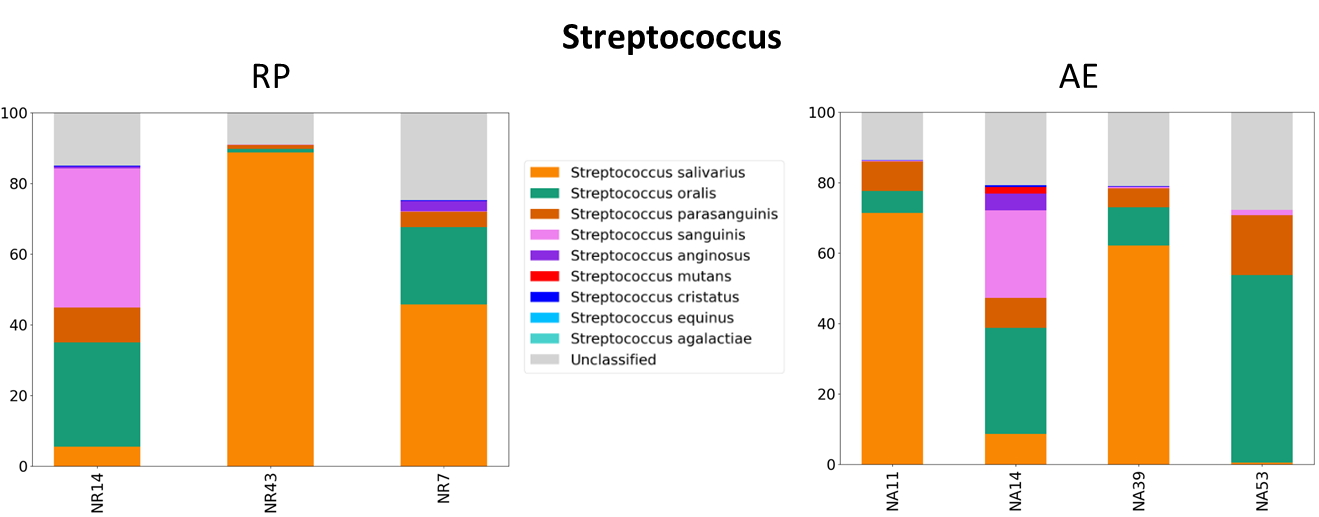


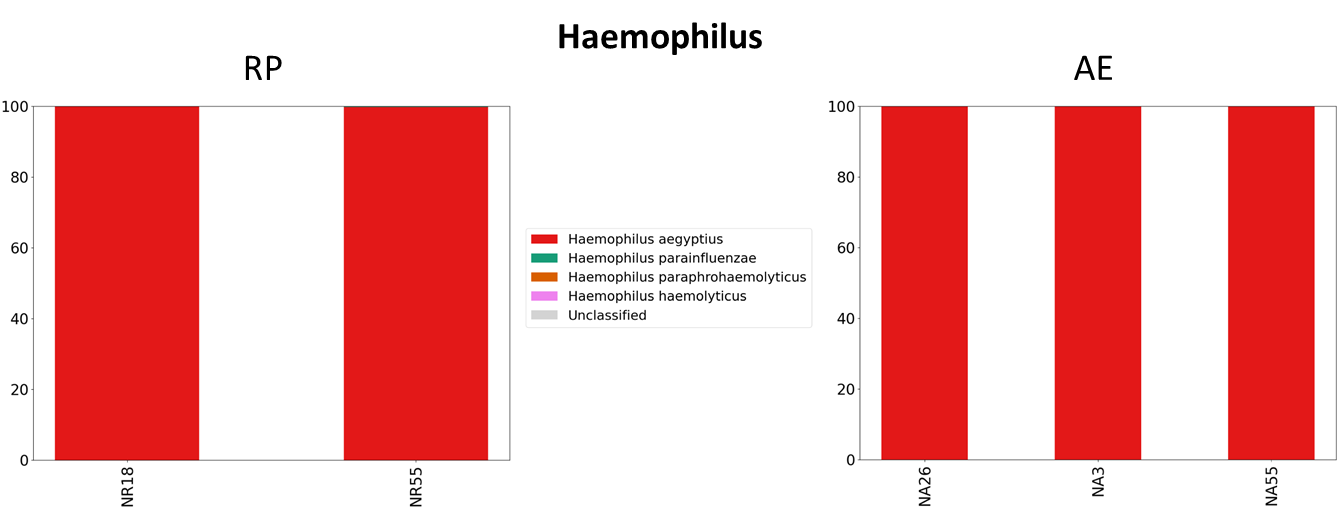


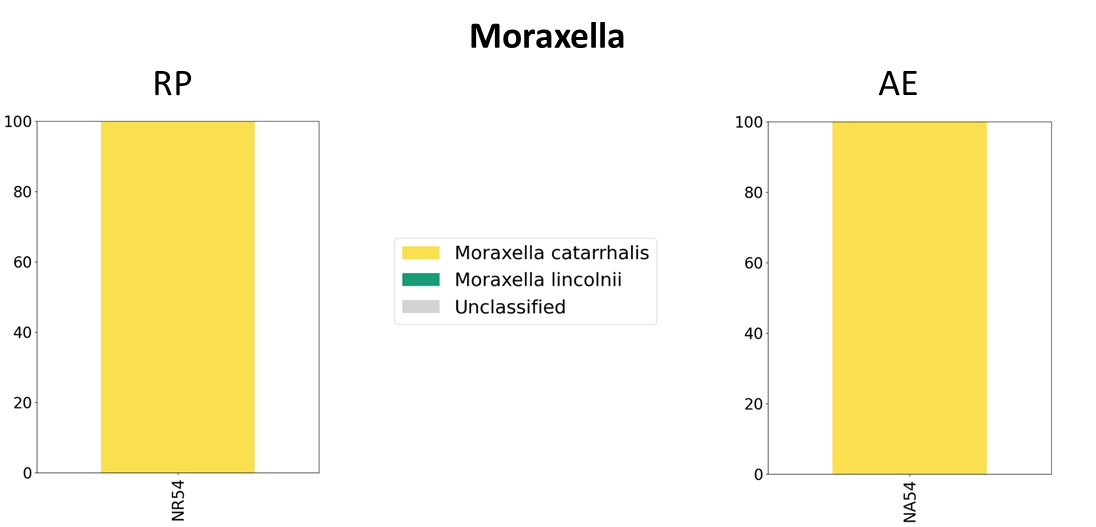


**Figure S1**. Species compositions of the six dominating genera in nasal microbiota. Classified species with a sum of percentages in all AE and RP samples greater than 0.8 were indicated while the rest are lumped together as unclassified.


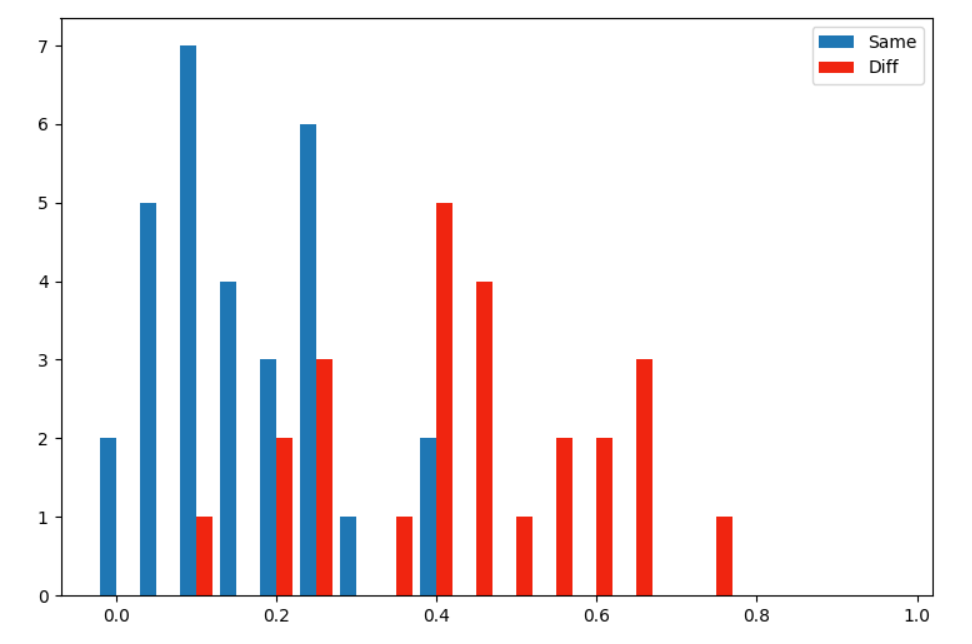


**Figure S2**. Distribution of weighted UniFrac distances between paired nasal microbiota that stay in the same microbial cluster (blue) or switch to a different cluster (red) when moving from AE into RP. The microbiota in paired samples are considered stable if the distance is <0.3.


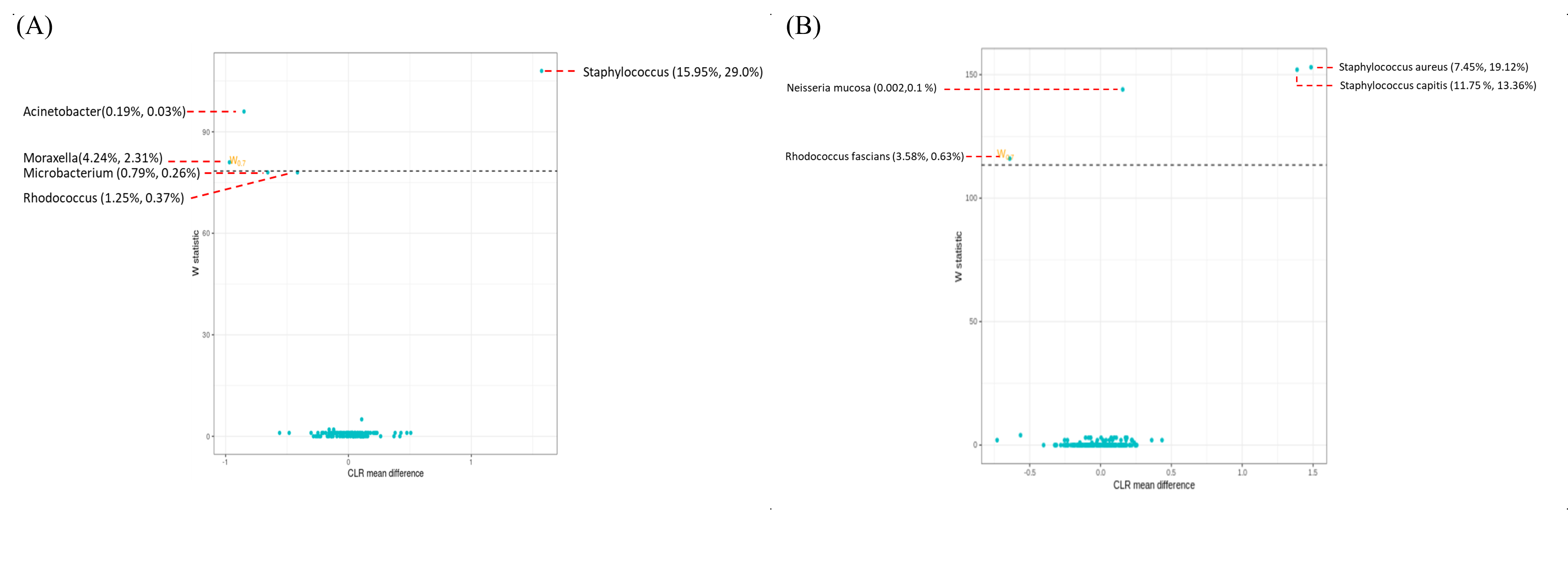


**Figure S3**. Pairwise differential abundance analysis of nasal microbes at the (A) genus level and (B) species level by ANCOM. Mean percentages of the significant taxons during AE and in the RP are indicated in the parenthesis.


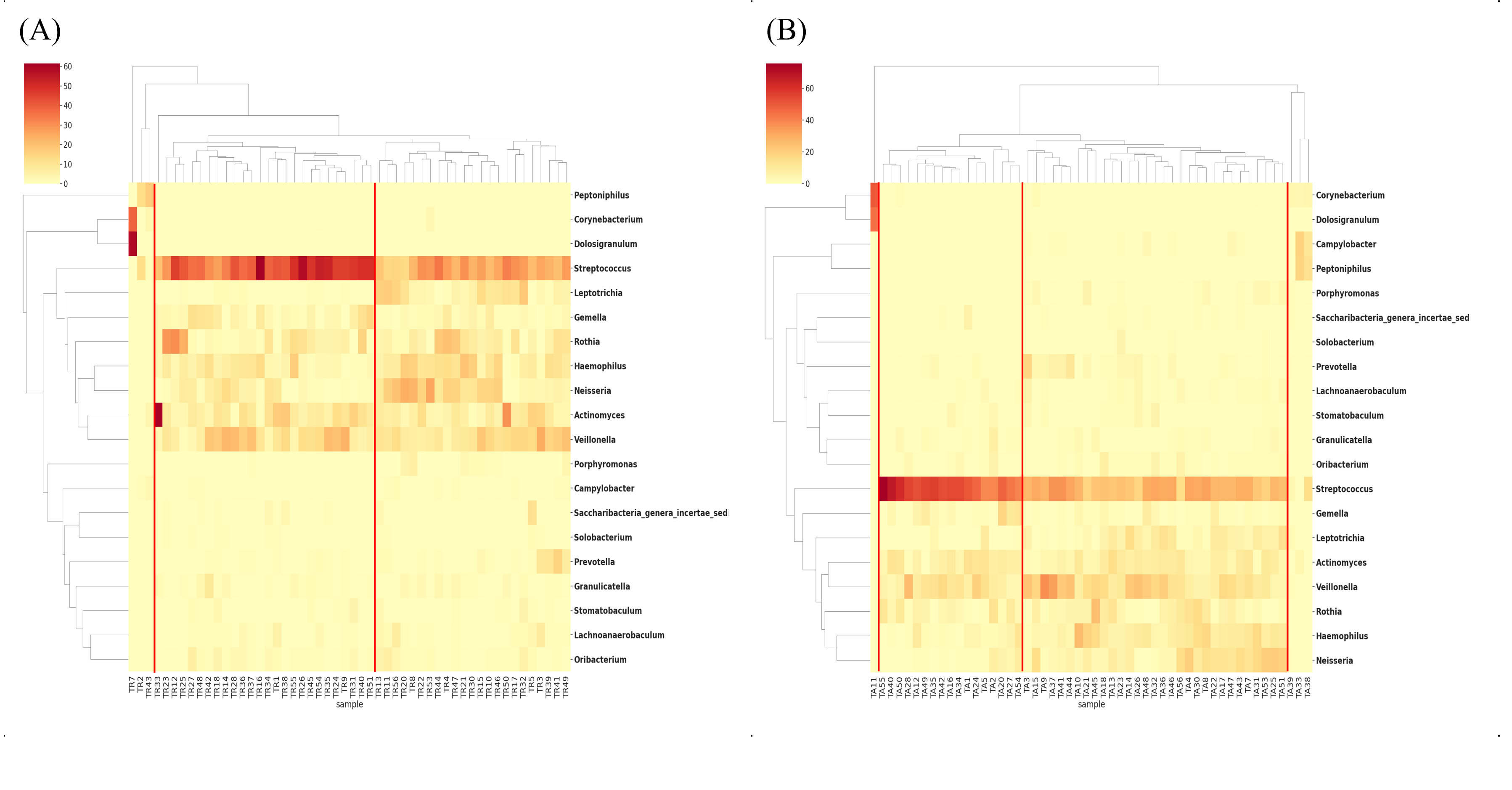


**Figure S4**. Throat microbiota (A) in the RP and (B) during AE at the genus level.

| (A)  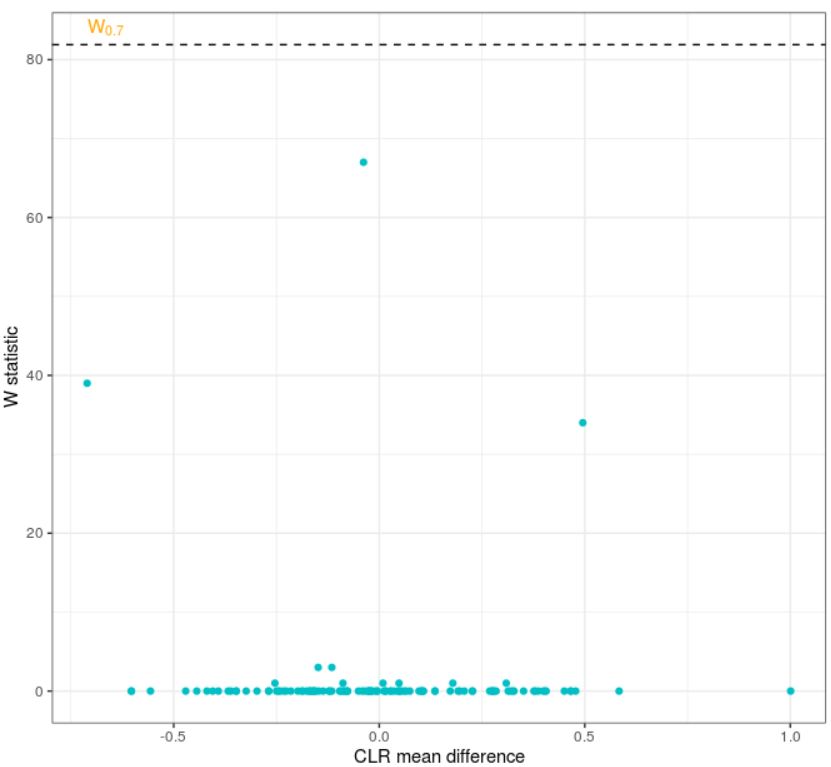 | (B)  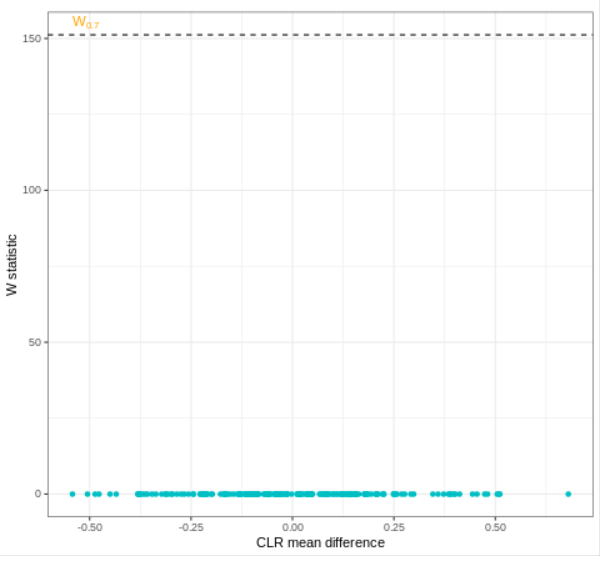 |
| --- | --- |

**Figure S5**. Pairwise differential abundance analysis of throat microbes at the (A) genus level and (B) species level.


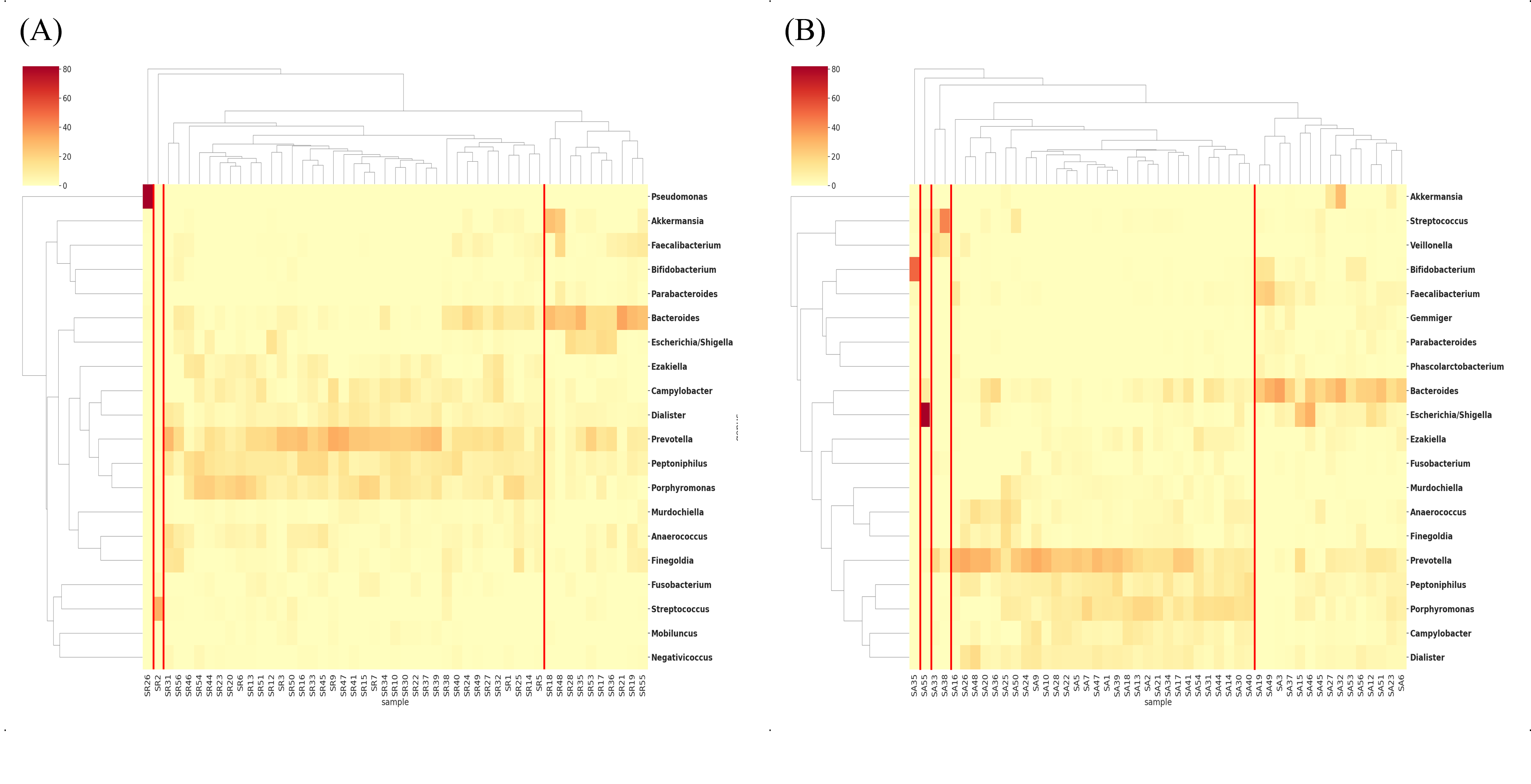


**Figure S6**. Stool microbiota (A) in the RP and (B) during AE at the genus level.


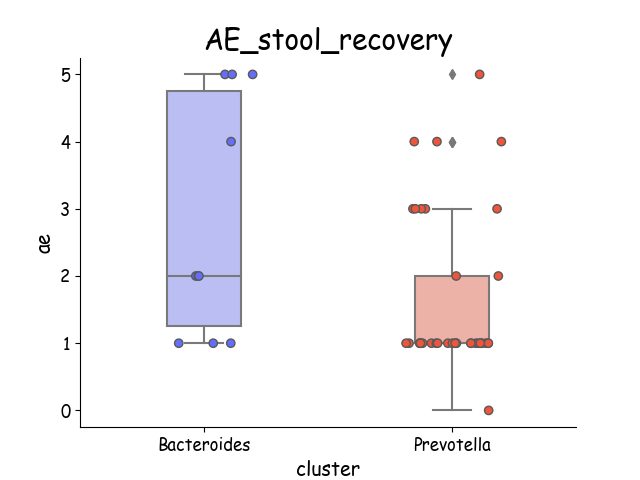


**Figure S7**. Subsequent AE frequency in the two RP clusters of stool samples.

| (A)  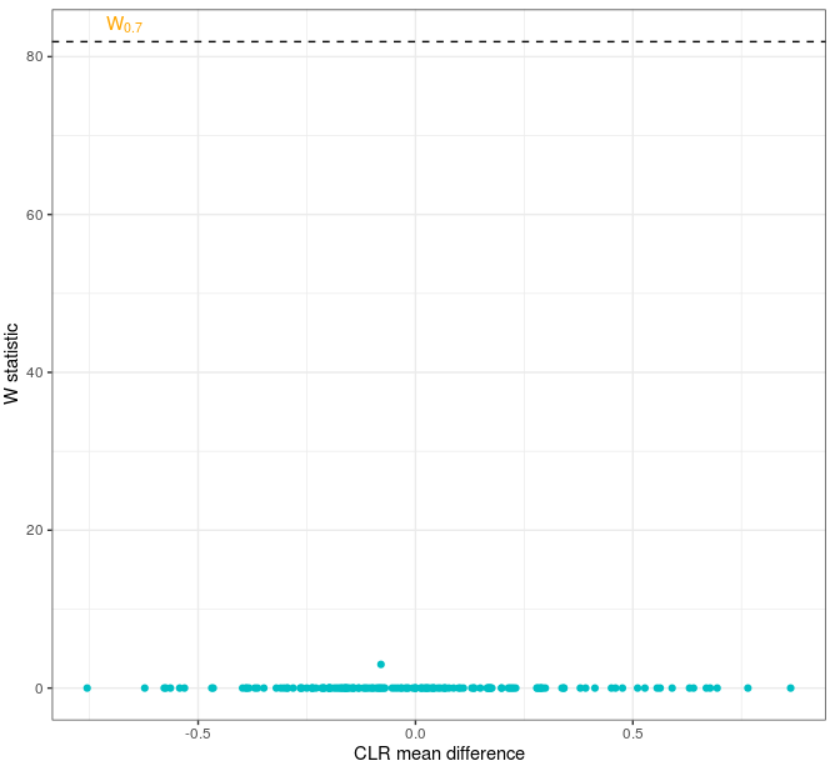 | (B)  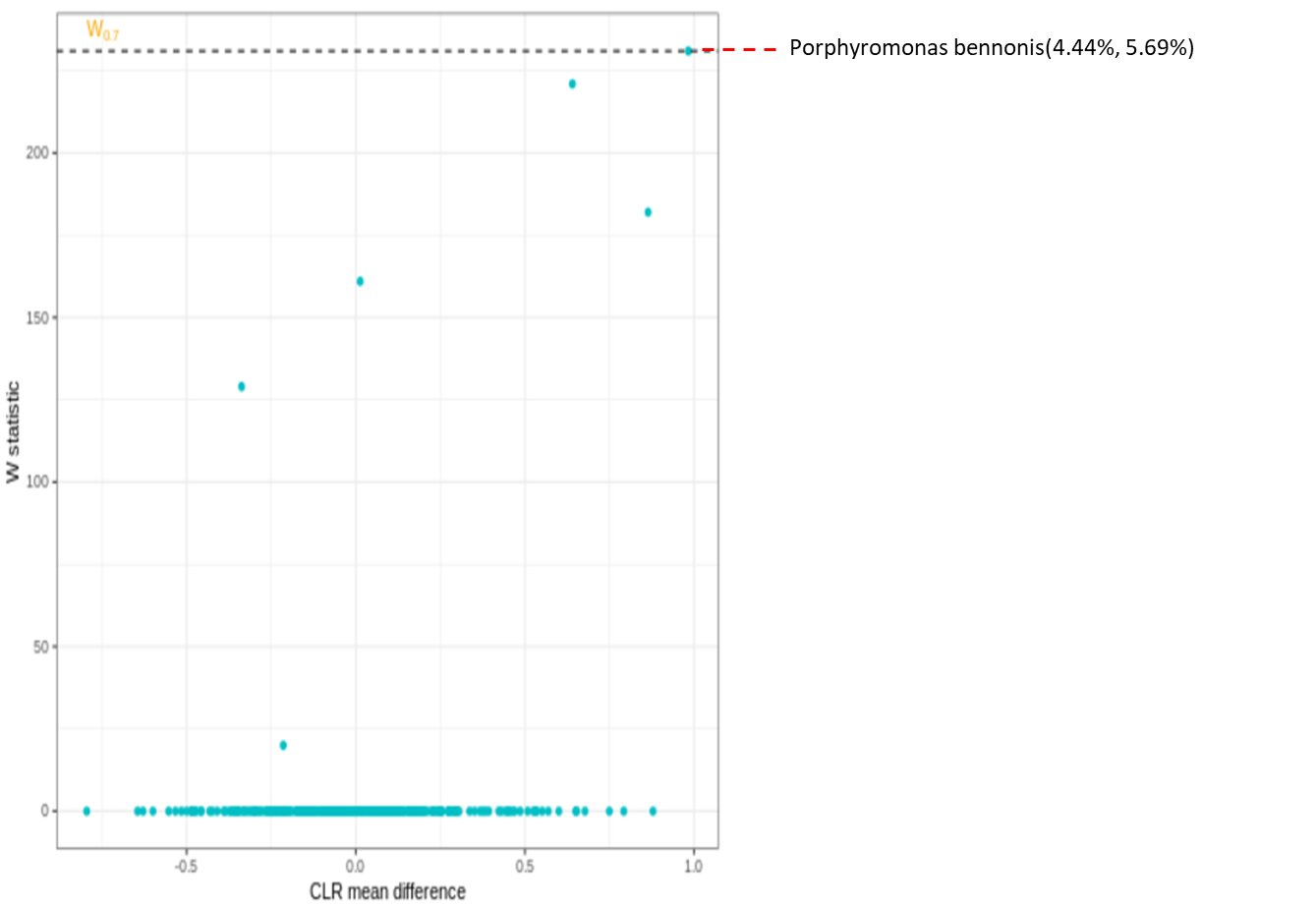 |
| --- | --- |

**Figure S8**. Pairwise differential abundance analysis of stool microbes at the (A) genus level and (B) Species level.
